# Outcomes and Lessons Learned From Immunization in Practice Training

**DOI:** 10.7759/cureus.108375

**Published:** 2026-05-06

**Authors:** Aeasha Karnik, Falah Wadi, Sumeet Juneja, Yahya Hasan

**Affiliations:** 1 Clinical Psychology, Chung-Ang University, Erbil, IRQ; 2 Health, United Nations Children's Fund (UNICEF) Iraq, Erbil, IRQ; 3 Epidemiology and Public Health, Immunization Supply Chain, Delhi, IND; 4 Immunization, Ministry of Health Holdings, Erbil, IRQ

**Keywords:** adult learning, capacity building programme, expanded programme of immunization (epi), immunization providers, immunization training, in-service training, kurdistan region of iraq, training quality assessment

## Abstract

Background

The Kurdistan Region of Iraq (KRI) faces persistent challenges in immunization service delivery due to workforce shortages, limited training opportunities, and unequal resource distribution. Most vaccinators lack formal training and rely on informal mentorship, often resulting in knowledge and skill gaps. In response, the Kurdistan Region Ministry of Health (KMOH), with support from the United Nations Children's Fund (UNICEF), implemented training sessions based on the World Health Organization (WHO) Immunization in Practice (IIP) guide to enhance vaccinators' competencies.

Objectives

This study aimed to (1) assess knowledge improvement following IIP training, (2) analyze participant feedback, and (3) evaluate the training modality against adult learning principles.

Methodology

A retrospective quantitative study design was applied to analyze 23 IIP training sessions conducted from October 2024 to January 2025 across Erbil and Sulaymaniyah Directorates of Health (DOHs). A total of 474 vaccinators participated. Data were collected through pre- and post-tests, feedback forms, and training observations. Quantitative data were analyzed using descriptive statistics and paired t-tests, while open-ended feedback and observations were used to provide contextual understanding of the findings.

Results

Immediate knowledge scores significantly improved in both DOHs. In Erbil, the average score increased from 7.32 to 11.52, equal to an improvement of +4.20 points. In Sulaymaniyah, the average score increased from 8.45 to 11.76, equal to an improvement of +3.31 points (p < 0.001). The participants' feedback was highly positive, especially regarding trainer performance. However, gaps were noted in content relevance, technical clarity, and training environment.

Conclusion

IIP training sessions enhanced vaccinators' immediate knowledge and were well-received. Future trainings should integrate more skill-based activities, improve logistical preparedness, and adopt a learner-centered approach to maximize impact and sustainability within the immunization workforce.

## Introduction

Service delivery, health workforce development, and health financing are three key building blocks of the health system out of the six as a strengthening framework proposed by the World Health Organization (WHO) [1]. Improving access to healthcare facilities and increasing immunization coverage require equitable, sustainable service delivery and a well-supported, efficient health workforce at each health facility.

The Kurdistan Region of Iraq (KRI) is a constitutionally recognized autonomous region located in northern Iraq. It comprises four governorates, namely, Erbil, Sulaymaniyah, Duhok, and Halabja, along with 29 districts [2]. The estimated population of KRI in 2023 was projected to reach 6,556,752 individuals. This includes 3,296,240 males and 3,260,514 females. Approximately 78.4% of the population resides in urban areas, while 21.6% live in rural areas. The annual population growth rate for 2023 was estimated at 2% [3].

KRI has a total of 1,482 health facilities, and the number of human resources (HR) under the Kurdistan Region Ministry of Health (KMOH) is 31,518 [2]. The Expanded Programme on Immunization (EPI) is provided in 344 health facilities; 132 in Erbil Directorate of Health (DOH), 119 in Sulaymaniyah DOH (including seven in Halabja), and 93 in Duhok DOH [4]. According to the KMOH data, the total number of HR dedicated to EPI is 805 health workers (291 in Erbil, 226 in Duhok, and 288 in Sulaymaniyah, including Halabja). Nevertheless, the health sector in KRI faces many challenges related to the three areas mentioned. It has affected healthcare providers' salaries, reduced the availability of training opportunities [5], created unequal distribution and shortage of HR [6-8]. Such challenges are likely to affect service quality in immunization, and the motivation of health workers, as well as poor immunization coverage [9]. HR newly assigned to immunization units usually do not receive pre-service formal training before taking on vaccinator roles. Rather, they follow and learn from their senior colleagues [10], who themselves may lack standardized training in immunization practices.

With limited resources available for capacity-building training, well-planned training design and content delivery are important to achieve quality and the fulfillment of desired objectives. The field of adult learning, known as andragogy, recognizes the unique aspects of adult learning, which differ from those of children, known as pedagogy. When these aspects are considered and integrated into training designs, they can lead to better learning outcomes [11-14]. Knowles et al. identified six interrelated core principles of adult learning: self-directed learning, prior experience, readiness to learn, problem-centered orientation, internal motivation, and the learner's need to know [12].

These principles have been incorporated into the design of training programs in the field of immunization [15-18]. To address the capacity-building gap, KMOH, with United Nations Children's Fund (UNICEF) support, took steps to enhance and update the knowledge of health workers by first translating the Immunization in Practice (IIP) guideline into Kurdish and distributing it to immunization units in health facilities. This guideline is a standardized, evidence-based practical resource followed internationally to improve immunization services [19]. Second, training sessions based on the guidelines were conducted across all districts in Erbil and Sulaymaniyah DOHs. This study aimed to evaluate the quality of the delivered training through changes in knowledge scores, participant feedback, and its alignment with adult learning methods.

Taking into consideration the mentioned development steps, the study was designed to achieve the following objectives:

Primary Objective

The primary objective is to assess the improvement in the knowledge of the vaccinators following the IIP training.

Secondary Objectives

The secondary objectives are to analyze participant feedback and to compare the training modality with adult learning principles.

## Materials and methods

Design

A retrospective quantitative study design was used, based on participants' pre- and post-test scores and training feedback. In addition, qualitative inputs, such as open-ended participant feedback, researcher observations, and non-structured individual and group discussions with trainers, EPI program managers, and participants during training sessions, were used to provide contextual understanding of the training. These qualitative inputs were not systematically coded or analyzed, but were used to support the interpretation of the quantitative findings.

Participants

The study population included health workers involved in immunization services across 8 districts in Erbil DOH and 12 districts in Sulaymaniyah DOH. The inclusion criteria included all the participants in the training. Participants were selected by the respective DOH based on their operational roles in immunization services, consisting of the majority of immunizers, along with one representative per primary health facility, as well as district EPI managers and cold chain supervisors for each training session. The exclusion criteria included anyone who was not a confirmed participant in the training. The years of experience in the immunization field varied among participants, with some being newcomers or volunteers, while others had long experience. The overall number of participants was 474, with 208 from Erbil and 266 from Sulaymaniyah.

Data collection

The main data collection tool used was a pre- and post-test, which was conducted on paper on the first day and was repeated at the end of the last day of the training. The test consisted of 15 questions that covered a range of essential topics related to vaccination practices. It included questions on technical knowledge, such as vaccine administration techniques, vaccine contraindications, timing of vaccination, and cold chain monitoring. Other questions assessed understanding of vaccine safety, open vial policy, and side effects. Programmatic aspects, such as dropout definitions, vaccine wastage calculation, and estimating vaccine need, were also covered. Additionally, the test evaluated interpersonal and communication skills through scenarios involving empathy, respectful behavior toward caregivers, and effective community engagement strategies. This variety of topics ensured both clinical competence and soft skills were thoroughly addressed in the training. Each question was given one point, and the passing threshold was set at 8 points. Responses from participants who missed either the pre- or post-test or did not record their identification number were excluded from analysis, leaving 452 valid responses, 186 from Erbil and 266 from Sulaymaniyah.

A training feedback form was also used, and it consisted of three sections. The first contained basic information about participants, including age, gender, years of experience, and perception of the training duration. The second section consisted of 12 Likert scale statements, rated on a 5-point scale from "totally disagree" to "totally agree", where higher scores indicated more positive feedback. The third section was an open-ended space for participants to provide suggestions for training improvement. Data collection in Erbil DOH was conducted on paper, resulting in some missing responses in the feedback form, especially in the participant's information section. Out of 208 participants initially enrolled, the number of responses varied by question (age: 190, gender: 202, years of experience: 201, perception of training duration: 203). In Sulaymaniyah DOH, electronic data collection using Kobo Toolbox was used to ensure no missing data. However, only 212 out of 266 participants filled the feedback form, due to their unfamiliarity with electronic data entry.

**Figure 1:**
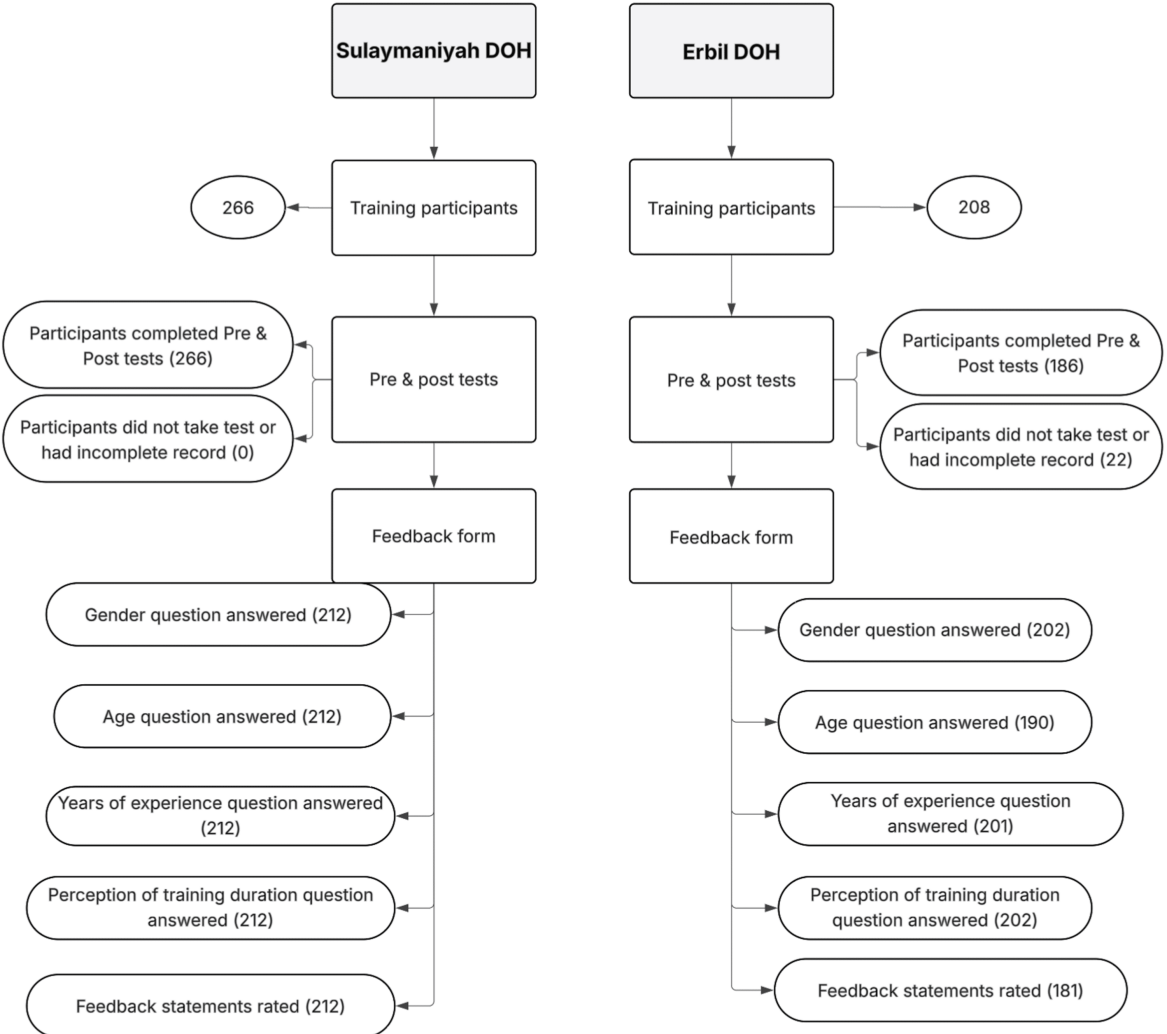
Flowchart of the data collection process DOH: Directorate of Health

The test and feedback forms were adapted from a previous IIP training package developed in Iraq, with the questions slightly adjusted to include only the topics covered in the KRI training. The forms were reviewed by the authors and the trainer and facilitator team, all of whom are EPI experts, to ensure relevance to the training context, and were then translated by the same team. However, no pilot testing was conducted before the training. The flow chart below (Figure 1) shows data collection details used for the analysis.

Training program

The training consisted of three days, conducted at several locations across both DOHs, with a total of 23 training sessions being conducted. Ten sessions were conducted in Erbil and 13 in Sulaymaniyah. The overall duration ran from October 13 to November 27, 2024, in Erbil, and from November 12, 2024, to January 9, 2025, in Sulaymaniyah. Each training had a team consisting of two trainers and two facilitators, along with two lecturers who delivered a single topic. A maximum of 25 participants was planned for each training; however, one to three additional attendees were accommodated in some sessions, except in the Soran training in Erbil DOH, which had 31 participants.

The training content was designed based on the WHO IIP guide and covered main topics, including an introduction to vaccination and the EPI program, the vaccine schedule in Iraq, vaccine safety and adverse events following immunization (AEFI), waste management, addressing disinformation and misinformation, improving communication skills, cold chain and vaccine management, estimating vaccine needs, vaccine contraindications, community engagement, missed opportunities, managing an immunization session, and vaccine data information management [19].

Training materials consisted of PowerPoint (Microsoft® Corp., Redmond, WA) presentation slides, flip charts, printed materials, data registries, and reporting formats. The presentation slides were in Arabic and English, while the paper materials were translated into Kurdish. In Sulaymaniyah DOH, parts of the PowerPoint slides were also translated into Kurdish. The training design was largely presentation-based, incorporating interaction with participants through questions and answers and sharing of experiences. Additionally, two group exercises (case study and vaccine need estimation calculation) were included over the course of the training. Some lectures were delivered in Arabic and translated by facilitators as needed. 

Data analysis

Microsoft Excel (version 16.94; Microsoft® Corp., Redmond, WA) was used to perform the analysis. Descriptive statistics, including mean and standard deviation, were used to summarize pre- and post-test scores. Change scores (post-test minus pre-test) were calculated for each participant, and the average improvement was calculated for each training session. A paired t-test was performed using individual paired pre- and post-test scores within each training session to assess whether the difference in knowledge scores was statistically significant. Results were summarized at the level of each training session and at the DOH level.

For the training feedback form, descriptive statistics, including mean, standard deviation, and percentage, were calculated for participants' basic information. In the feedback section, the mean and standard deviation were calculated for each statement, along with the total feedback scores. Suggestions provided by participants in the open-ended question were summarized by grouping similar responses to identify recurring suggestions.

Participants with missing pre- or post-test, or missing identification numbers on one or both tests, were excluded from the analysis. For the feedback form, the first and third sections, which included basic participant information and suggestions, were analyzed based on available responses.

Ethical considerations

All the data collected from these training sessions were originally collected for the purpose of training evaluation, which was later used in this retrospective study. No personal information about the participants was collected through tests or feedback forms. This study was approved by the Ministry of Health Scientific Research in Erbil.

## Results

Participants information

The majority of participants in both Erbil (84%) and Sulaymaniyah (86%) DOHs were female. The highest represented age groups were 40-49 years and 50-59 years, with a mean age of 45 years (SD = 0.68) in Erbil and 44 years (SD = 0.67) in Sulaymaniyah. Most participants had more than five years of experience (63% in Erbil, 66% in Sulaymaniyah), as can be seen in Table 1. 

**Table 1:** Participants' information DOH: Directorate of Health

Participants' information	Erbil DOH	Sulaymaniyah DOH
	Distribution, n (%)	Distribution, n (%)
Sex		
Male	72 (36)	29 (14)
Female	130 (84)	183 (86)
Total answers	202	212
Age		
20-29	17 (9)	24 (11)
30-39	33 (17)	40 (19)
40-49	67 (35)	63 (30)
50-59	68 (36)	81 (38)
>=60	5 (3)	4 (2)
Total answers	190	212
Years of experience		
From one to five years	48 (24)	59 (28)
More than five years	127 (63)	139 (66)
Less than a year	26 (13)	14 (7)
Total answers	201	212
Perceived training period		
Long	40 (20)	41 (19)
Not too long nor too short	142 (70)	163 (77)
Short	20 (10)	8 (4)
Total answers	202	212

Test scores

A total of 10 IIP training sessions were conducted in Erbil and 13 in Sulaymaniyah, covering all districts. Training sessions in both DOHs resulted in a significant improvement in participants' post-training scores, as shown in Table 2. 

**Table 2:** Pre- and post-test scores Note: Passing score = 8; total possible score = 15 DOH: Directorate of Health; Tr: Training

DOH	Pre-test, Mean (SD)	Post-test, Mean (SD)	Improvement mean (SD)	P value
					Erbil DOH training sessions				
Tr 1: Erbil Center	8.16 (2.24)	10.74 (2.45)	2.58 (2.67)	< 0.001
Tr 2: Erbil Center	7.35 (3.08)	11.78 (2.02)	4.43 (2.25)	< 0.001
Tr 3: Khabat	6.60 (3.87)	12.60 (1.64)	6.00 (3.38)	< 0.001
Tr 4: Khabat	8.25 (2.60)	11.42 (1.56)	3.17 (2.04)	< 0.001
Tr 5: Dashti Hawler	7.84 (2.03)	11.79 (1.81)	3.95 (2.48)	< 0.001
Tr 6: Dashti Hawler	7.50 (2.09)	11.75 (1.62)	4.25 (1.65)	< 0.001
Tr 7: Koya	6.89 (3.62)	11.21 (2.23)	4.32 (3.27)	< 0.001
Tr 8: Shaqlawa	6.16 (2.76)	11.58 (2.55)	5.42 (3.01)	< 0.001
Tr 9: Soran & Choman	6.81 (3.09)	10.88 (2.86)	4.08 (2.50)	< 0.001
Tr 10: Barzan	8.21 (2.81)	11.86 (3.88)	3.64 (4.48)	0.009
Total	7.32 (2.88)	11.52 (2.35)	4.20 (2.87)	< 0.001
Sulaymaniyah DOH training sessions				
Tr 1: Sulaymaniyah center	6.13 (4.22)	10.17 (3.16)	4.04 (2.93)	< 0.001
Tr 2: Sulaymaniyah center	7.63 (2.79)	11.5 (2.73)	3.88 (1.68)	< 0.001
Tr 3: Sulaymaniyah center, Sharbarez, Dokan, Shahrazur & Darbandikhan	9.64 (2.84)	12.27 (2.98)	2.64 (1.50)	< 0.001
Tr 4: Sulaymaniyah center, Sharbarez, Dokan, Shahrazur & Darbandikhan	9.54 (3.41)	12.42 (2.95)	2.88 (2.44)	< 0.001
Tr 5: Ranya	7.11 (3.02)	11.84 (1.95)	4.74 (2.13)	< 0.001
Tr 6: Sulaymaniyah center	8.73 (2.86)	11.73 (2.52)	3.00 (1.70)	< 0.001
Tr 7: Ranya	7.65 (3.25)	10.6 (2.14)	2.95 (2.65)	< 0.001
Tr 8: Halabja Shahid, Said sadiq & New Halabja	8.57 (3.37)	12.14 (3.06)	3.57 (1.99)	< 0.001
Tr 9: Sulaymaniyah center	9.16 (2.93)	12.04 (2.49)	2.88 (2.42)	< 0.001
Tr 10: Chamchamal & Bazyan	9.57 (2.79)	12.29 (2.46)	2.71 (1.86)	< 0.001
Tr 11: Garmyan 14	9.625 (2.62)	12.42 (2.28)	2.79 (2.64)	< 0.001
Tr 12: Chamchamal & Bazyan	9.21 (3.38)	12.14 (3.06)	2.93 (2.46)	< 0.001
Tr 13: Halabja Shahid, Said Sadiq & New Halabja	7.44 (2.97)	11.50 (2.78)	4.06 (1.65)	< 0.001
Total	8.45 (3.27)	11.76 (2.71)	3.31 (2.27)	< 0.001

The pre- and post-test scores show that the training sessions in both Erbil and Sulaymaniyah had a positive impact on participants' knowledge. Across all 10 training sessions in Erbil, participants improved significantly. The average pre-test score was 7.32, which increased to 11.52 after the training. This means that, on average, participants gained about 4.2 points in knowledge. The most significant improvement was seen in Khabat (Training 3) with a gain of 6 points. Even the lowest improvement, 2.58 points in Erbil Center (Training 1), was still statistically significant. All sessions had a p-value of < 0.001, except Barzan (Training 10), which still showed great improvement with p = 0.009.

The 13 training sessions in Sulaymaniyah also showed consistent improvement. The average pre-test score was 8.45, which rose to 11.76 after training, an average gain of 3.31 points. The greatest improvement was in Ranya (Training 5) with a 4.74-point gain. The lowest average gain was around 2.64-2.88 points in a few sessions, still showing solid learning progress. All sessions had a p-value of < 0.001, confirming the improvements were statistically significant.

Feedback statements

**Figure 2:**
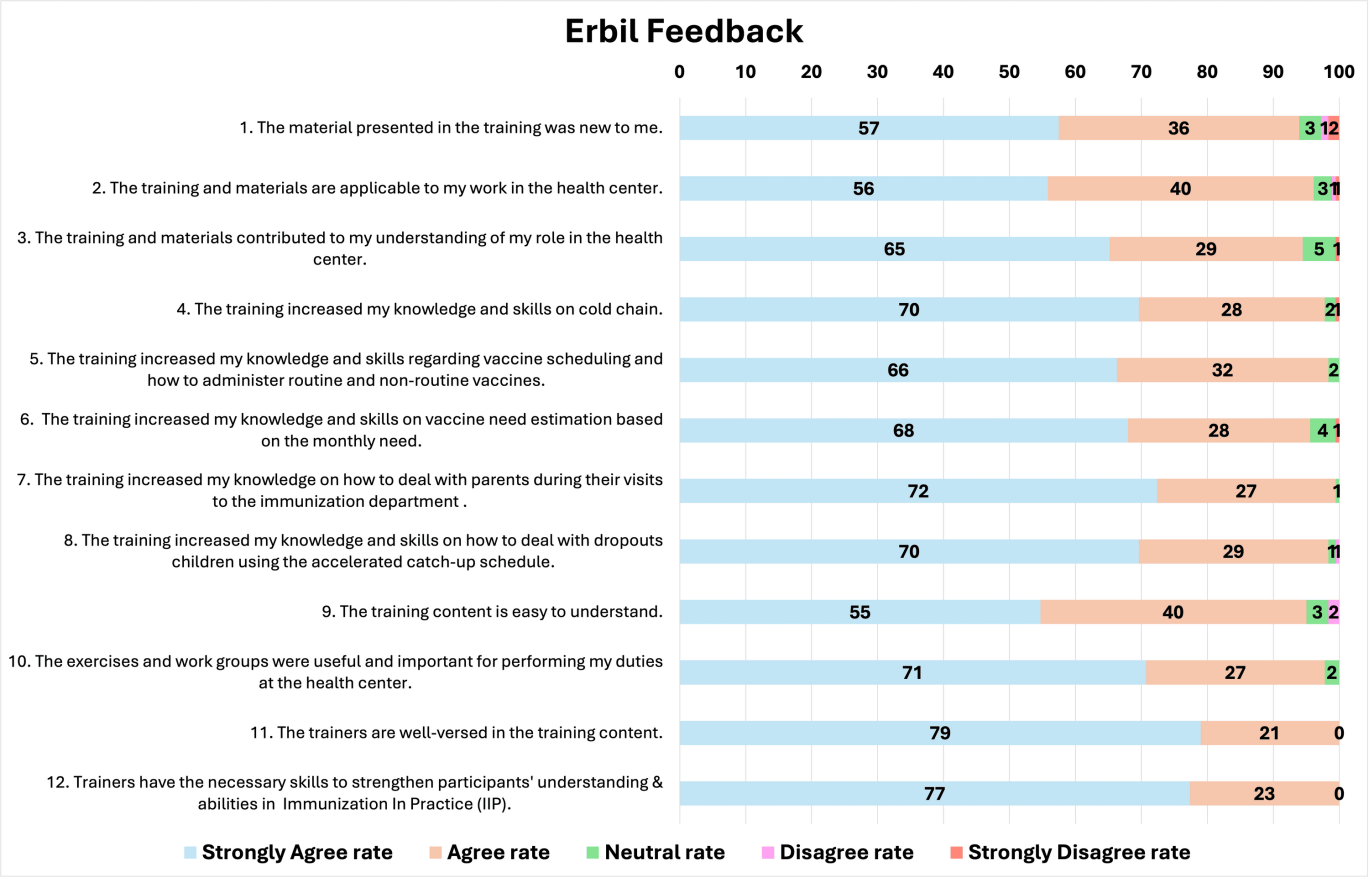
Participants' feedback from Erbil

**Figure 3:**
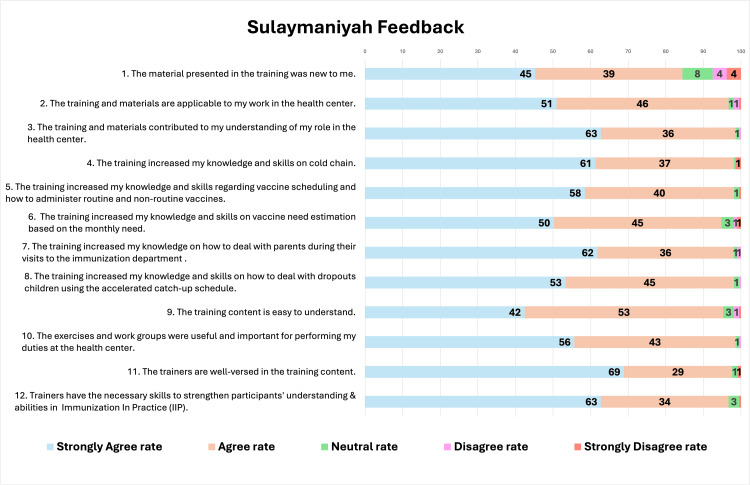
Participants' feedback from Sulaymaniyah

The overall training feedback was highly positive, as most responses were categorized as "strongly agree" and "agree" for all statements. However, some participants provided "neutral" or "disagree" responses, particularly for Statements 1, 2, 3, 6, and 9 in Erbil and Statements 1, 6, and 9 in Sulaymaniyah. In addition, participants in both Erbil and Sulaymaniyah consistently rated the trainers positively, especially in terms of their knowledge and ability to strengthen understanding (Statements 11 and 12). On the other hand, lower agreement levels were observed for statements related to content novelty, clarity, and calculation-based tasks (Statements 1, 6, and 9). Figures 2-3 present the percentage of responses for each statement and highlight aspects of the training that require further attention. Regarding training duration, the majority of participants, 70% in Erbil and 77% in Sulaymaniyah, considered the duration to be appropriate, as shown in Table 1.

Participants' suggestions for training improvement

Participants used the open space to leave positive comments and express their appreciation for the training opportunity, with many requesting periodic capacity development training sessions, especially on immunization micro-planning. Additionally, several participants requested greater support from the Erbil DOH in enhancing support across various aspects of their work, particularly in increasing the number of health workers in the immunization unit. However, only a few participants provided specific suggestions for improving future trainings, which are summarized in Table 3.

**Table 3:** Participants' suggestions for training improvement

Freq. of responses	Suggestions for Erbil trainings	Freq. of responses	Suggestions for Sulaymaniyah trainings
3	Providing a manikin to properly show vaccine injection positions for children.	2	The sessions conducted in Arabic, followed up with Kurdish translation, took up a lot of training time. It would be easier to understand if all lectures were presented in Kurdish.
1	Incorporating video materials into the various topics, including vaccine administration techniques.	1	Adding skill practice sessions to the training.
1	Conducting all the sessions in Kurdish to make understanding easier.	3	Extending the total duration of the training and concluding each day by 12 PM.
7	Extending training duration and including a session on micro-planning.		

## Discussion

The IIP training conducted through this modality in the studied sample of both Erbil and Sulaymaniyah has successfully increased vaccinators' knowledge about various aspects of EPI as an immediate outcome. The first objective of this study was achieved in improving the knowledge of vaccinators in standardized immunization practices. Specifically, this type of comprehensive training in immunization was conducted for the first time in KRI.

Similar outcomes were found in studies that used training interventions with content partially similar to this study, such as the study by Uskun et al. [20] in Turkey, which also reported a significant increase in primary healthcare workers' immunization knowledge, as reflected in post-test scores following a three-day workshop, as well as an increase in vaccination coverage rates in the three months after the intervention. Other studies in Nigeria used experimental and quasi-experimental designs to test their training intervention. In a study [21], assessments were conducted at pre-intervention, immediately after the training, and at three and six months. Knowledge improvements were observed at their highest immediately after the training, attributed to the effective and diverse training methods. However, a drop in the mean score after three and six months of the training was noted. In Ameen et al.'s study, assessments were conducted at baseline, three months, and six months after intervention [22]. A statistically significant improvement in knowledge and practices was observed at three months, followed by a decline at six months.

Immunization-related tasks in vaccination units in KRI are usually distributed between the health workers, with vaccinators primarily responsible for administering vaccines, with limited interference with other immunization duties, such as cold chain management and vaccine need estimation. This gap in practice highlights the significance of the IIP training in expanding vaccinators' understanding of the EPI aspects.

Notably, the findings also showed that the majority of vaccinators in both DOHs belonged to an older age group. This observation indicates a possible weakness in the KRI's health system, where new cadre recruitment is nearly absent. This might lead to a significant gap when the older age group of vaccinators retires, resulting in shortages of immunization staff or possibly the closing of some immunization units. Furthermore, the immunization units are often undesired by the HR due to their heavy workload and multiple responsibilities, which hinder volunteers and newly appointed staff from transferring to these units.

Participants provided very positive feedback on the training, and the duration of the training was mostly considered appropriate. However, certain feedback statements received few "neutral" or "disagree" responses. Although minimal, these responses can provide insights into some training aspects requiring further attention.

As most participants had over five years of experience, 6% in Erbil and 16% in Sulaymaniyah did not fully agree with the statement "The material presented in the training was new to me." Similarly, responses to the second and third statements, which assessed the applicability of the training to participants' work and its contribution to their understanding of their role, showed that 5% and 6% of participants in Erbil expressed neutral responses or disagreement. This may indicate a reluctance to fully recognize how relevant the training content is to their routine tasks.

Observations during training sessions further clarified this reaction, as some participants were skeptical about the feasibility of fully practicing all immunization tasks and demands in their work. The most challenging elements to apply in their point of view included using standard vaccine need estimation to calculate vaccine need and request a number accordingly, completing all nine required registries regularly, tracking defaulters and conducting outreach sessions, and communicating and providing awareness to all parents. The main barriers to compliance with these practices were identified as staff shortages, workload pressure, and insufficient financial support.

Furthermore, several participants were observed to be disengaged in each training session in both DOHs. They showed a lack of passion for their role as vaccinators and a limited interest in improving their performance.

The participants showed uncertainty about their knowledge and skills in vaccine need estimation, as reflected in 5% of responses to Statement 6 from both DOHs. This subject required the most time and effort during training, with many participants finding it challenging, as it was contrary to their usual practice of determining vaccine need based on personal estimates and lacked consistent record-updating practices needed for accurate estimation. The training revealed this critical gap in knowledge related to practices in KRI that may contribute to vaccine wastage or vaccine shortages in health facilities due to miscalculations in need estimation or inadequate reserve stock levels. Furthermore, many vaccinators, despite their long experience in their designated health facility, were unaware of their monthly target when asked.

Regarding participants' feedback statement on the training content being easy to understand, 5% of participants in Erbil and 4% in Sulaymaniyah found it challenging to understand. Even though several factors may have contributed to this, participants' suggestions in Table 4 (e.g., incorporating practical sessions, demonstrations, and instructional videos) highlight a training weakness, which was mostly lecture-based. More interactive activities could have enhanced content understanding.

Language barriers also created challenges, as some participants struggled to follow Arabic sessions and suggested that all content be presented in Kurdish. While translation was provided, PowerPoint slides in Erbil remained in Arabic; however, in Sulaymaniyah, translations were gradually added after initial feedback from participants.

The IIP training showed several strengths aligned with key adult learning principles. The design and content met the *Readiness to Learn* principle by being highly relevant to the roles of participants. The orientation of learning was mostly "Problem-Centered," as it included case studies and provided real-life examples and applicable solutions to work-related challenges. The "Prior Experience" of the participants was also considered; the sessions enhanced their knowledge through experience sharing and group tasks.

Furthermore, the training showed some flexibility by adapting to the limitations of the learning environment and addressing the knowledge gaps of the participants. The diversity of the content, which was delivered by six facilitators with varying presentation styles, helped make the sessions more interesting.

The training also targeted "Internal Motivation" of participants by highlighting the significance of the vaccinator's role in society and acknowledging their commitment to providing service despite various challenges. At the end of the training, completion certificates were distributed as a means of enhancing motivation.

On the other hand, several weak points were noted, some of them may have conflicted with adult learning principles. Due to the training sessions being conducted in various locations, the quality of the learning environment differed significantly. In some training halls, where the infrastructure was not suitable for more than 15 people, the number of participants exceeded 25, resulting in overcrowding. Logistical issues due to the unavailability of basic electronic equipment in some halls and a lack of preparation hindered smooth delivery.

Furthermore, a lack of preparation affected the start time of many training sessions, where proper training introduction and self-presentations were either absent or rushed. Inability to provide time for the participants to have a complete understanding of the training's purpose, objectives, expectations, and rules conflicts with the "Need to Know" principle and may have diminished motivation and readiness to learn.

On some occasions, the learning environment was similar to a traditional classroom, promoting a "dependent" learning role in participants. Noise and limited control over the training environment were also noticeable challenges. Additionally, the main facilitators were not present during the full training period, which affected group activities and limited support for learners. Distributing participants into groups was also a limitation; in certain training sessions, groups had 10 participants, making engagement or keeping up with discussions difficult for many of them. Even with the presence of many facilitators, attempts to engage inactive participants were minimal. Failure to address these points leads to a clear disparity in participants' reaction to the training, with some being motivated and actively involved, while others remained silent and inactive, and a few were distracted and disengaged.

The different presentation style of facilitators revealed some weakness in body language and nonverbal communication skills, which unintentionally excluded certain participants, for example, those seated at the sides of a long U-shaped format, or those seated in the back.

Based on these reflections, the following points are suggested to consider in future training sessions to enhance quality and learning outcomes:

(1) Include a planned introduction session in the training, by outlining the learning objectives that are expected to be met by the end of the training. This step helps in reducing the anxiety related to the unknown, enhances attention, and increases the likelihood of achieving desired outcomes [23]. It is also essential to review the agenda and establish training rules at the beginning. Additionally, an icebreaker activity is recommended to create a welcoming, adult-friendly learning environment where all participants feel included.

(2) The lecture parts of the training can be strengthened by incorporating interactive activities, such as Q&A or short discussions at specific points to stimulate knowledge recall, support attitude change, and reduce resistance to learning [23].

(3) Extend the goal beyond increasing participants' knowledge to also fostering behavioral change through incorporating more skill development components to the training, such as demonstrations followed by practice time. It is important to consider small group sizes to ensure meaningful participation for all within time limits; a ratio of one trainer per five participants is considered a rule of thumb for group-based exercises [23]. Behavior modeling technique can be used to develop proper vaccine administration skills, an area noted to be lacking among many vaccinators during the training. Thus, the first step is to show a standardized administration model via video, followed by a discussion on correct and incorrect practices. Then, each participant within small groups practices the technique using a manikin, under the observation and feedback of facilitators [23].

(4) With limited learning resources, facilitators need to be well-prepared to address expected challenges, such as having additional electronic equipment. Facilitators' teamwork and their full presence are also important to effectively manage the learning environment.

(5) Use digital data collection tools for forms and tests to minimize data loss.

(6) Plan post-training follow-up visits to vaccination units to promote implementation of knowledge and to assess field practice and immunization outcomes, as knowledge may wane if not reinforced with supportive supervision, monitoring, and regular training [22]. Erbil DOH took an initial step after the conclusion of the training sessions by issuing an official letter to all immunization units to comply with immunization standard practices. However, a follow-up plan or evaluation was not conducted. The district EPI managers can utilize their routine supervision visits to reinforce the application of learned content and provide on-the-job guidance.

Furthermore, to achieve the desired immunization outcomes in practice, the health system in KRI needs to take active steps to improve support for HR. This includes ensuring consistent salaries, increasing HR in immunization units, and providing a communication card and transportation fee for outreach sessions. Finally, strengthening routine training and incorporating these lessons into national immunization capacity-building strategies can improve system-wide performance.

## Conclusions

The IIP training sessions conducted in Erbil and Sulaymaniyah DOHs were successful in providing content that addressed the needs of participants and enhanced their immediate knowledge about standardized immunization practice, demonstrated in their post training scores and high feedback ratings. While the training met some aspects of adult learning, it also revealed some weaknesses in training delivery format, management of the learning environment, and active facilitation and learner support. Key learned lessons include engaging all participants, incorporating skill-building components, and encouraging implementation through post-training follow-up.

This study includes several limitations, which are limited generalizability beyond the Erbil and Sulaymaniyah context, the possibility of self-report and social desirability biases in the feedback form, and not piloting the adapted and translated tools prior to the training. Qualitative inputs were mostly non-structured and were not systematically analyzed, but were used to support the interpretation of the findings. Finally, the study did not assess long-term retention of knowledge or changes in workplace practice, so the findings are limited to immediate training outcomes. Further research would be needed to assess how knowledge changes translate into practice.
